# Psychopathic Hospitals

**Published:** 1915-03

**Authors:** 


### ABSTRACTS

**Psychopathic Hospitals,** F. S. White, Texas Medical Journal, January 1915.

Average Recovery Rate, Death Rate and Per Capita Cost.

| | Number Hospitals | Recovery Rate. | Death Rate. | Per Capita Cost. |
|---|---|---|---|---|
| Arizona | 1 | 31.19 | 8.00 | $370.92 |
| Arkansas | 1 | 44.00 | 6.84 | 130.00 |
| California | 5 | 26.03 | 5.89 | 175.63 |
| Connecticut | 2 | 22.57 | 8.46 | 184.96 |
| Delaware | 1 | 23.71 | 12.00 | 157.04 |
| Florida | 1 | 20.09 | 9.84 | 197.40 |
| Georgia | 1 | 6.30 | 11.75 | 132.47 |
| Illinois | 6 | 26.11 | 6.28 | 157.64 |
| Indiana | 3 | 23.04 | 6.74 | 192.65 |
| Iowa | 1 | 31.00 | 6.00 | 197.00 |
| Kentucky | 3 | 24.84 | 8.45 | 151.94 |
| Kansas | 2 | 39.11 | 10.00 | 164.34 |
| Louisiana | 1 | 14.00 | 9.80 | 121.32 |
| Maine | 1 | 18.60 | 10.70 | 192.80 |
| Massachusetts | 5 | 14.05 | 8.42 | 235.84 |
| Maryland | 1 | 24.00 | 4.70 | 203.00 |
| Michigan | 2 | 27.10 | 5.64 | 179.77 |
| Minnesota | 4 | 23.20 | 9.00 | 123.97 |
| Mississippi | 1 | 24.00 | 6.30 | 150.00 |
| Missouri | 4 | 25.40 | 8.44 | 167.38 |
| Nebraska | 1 | 21.30 | 9.00 | 232.00 |
| Nevada | 1 | 47.00 | 16.66 | 193.00 |
| New Mexico | 1 | 26.10 | 12.95 | 186.97 |
| New York | 10 | 30.90 | 5.96 | 201.12 |
| Ohio | 5 | 26.92 | 7.38 | 153.99 |
| Oregon | 1 | 66.00 | 10.25 | 164.52 |
| Pennsylvania | 2 | 24.43 | 6.65 | 215.40 |
| Tennessee | 2 | 15.16 | 8.75 | 142.50 |
| Utah | 1 | 33.00 | 7.60 | 146.00 |
| Virginia | 2 | 13.91 | 7.77 | 132.97 |
| Washington | 1 | 31.92 | 8.05 | 170.55 |
| Wyoming | 1 | 44.00 | 4.80 | 188.51 |
| | 74 | 26.84 | 8.70 | $180.11 |
| Texas | 3 | 40.80 | 5.77 | $164.89 |
| S. W. Insane Asylum | .. | 50.00 | 7.22 | 159.27 |

